# Supplementary material for: Farmers’ Views and Tools Compared with Laboratory Evaluations of Parasites of Meat Goats in French West Indies
Source: Animals (Basel). 2023 Jan 26;13(3):422. doi: 10.3390/ani13030422 (PMC9913335; doi:10.3390/ani13030422)
Supplement: Supplementary file 1 [file animals-13-00422-s001.zip › animals-2114284-Supplementary File S1.pdf]

Guide for open questions to Guadeloupe goat farmers

1) How would you describe your farm?
Do you do livestock only or livestock and crop? Only goats or other animals? Is this your main activity? How many workers do you have? How many animals? What breeds of goats?

2) How did you get into goat keeping?
How long have you been doing it? What experience did you have? Your background: how did you trained? Was it a family takeover? Reconversion?

3) What problems do you encounter?
Feeding the goats? Goat diseases? Accidents? Fatigue and difficulty of work?

4) If parasitism (external, internal) has not yet been mentioned
Is it a complicated problem or not? How do you spot it?

5) In case of pathology, what do you do?
Who do you turn to? Why? How do you treat it?

6) What do you like about your work? What do you dislike?

7) What is good work? And bad work?
Having a herd that makes money? A healthy herd? Doing your own breeding?

8) How do you feel about selling your animals?
Do you have pain? Are you joyful? Is it difficult? Did you develop attachment to the goats?
